# Effect of CaCl_2_ on the Structure of DODAB Vesicles Containing the Ganglioside GD3

**DOI:** 10.1021/acsomega.6c03945

**Published:** 2026-08-17

**Authors:** Julia B. Ejarque, Evandro L. Duarte, M. Teresa Lamy, Julio H. K. Rozenfeld

**Affiliations:** † Departamento de Biofísica, Escola Paulista de Medicina, Universidade Federal de São Paulo, R. Botucatu 862, São Paulo, São Paulo 04023-062, Brazil; ‡ Instituto de Física, 61755 Universidade de São Paulo, Rua do Matão 1371, São Paulo, São Paulo 05508090, Brazil

## Abstract

CaCl_2_ has been successfully used in the formulation and stabilization of vaccines. Vesicles made of the disialoganglioside GD3 and the cationic lipid DODAB are promising immunotherapeutic tools, by combining the targeting properties of GD3 and the adjuvant properties of DODAB. In this work, the effects of CaCl_2_ on the structure of DODAB and DODAB+GD3 vesicles have been compared by Differential Scanning Calorimetry (DSC) and Electron Paramagnetic Resonance (EPR) spectroscopy. EPR spectra show that CaCl_2_ does not induce the formation of GD3 domains by lateral phase separation. DSC thermograms show that CaCl_2_ hinders the formation of a subgel phase for vesicles cooled below 15 °C. CaCl_2_ also decreases the hysteresis between gel–fluid and fluid–gel transitions in both pure and mixed vesicles. EPR data show that CaCl_2_ increases the superficial rigidity of DODAB and DODAB+GD3 vesicles at temperatures below the gel–fluid transition. In this temperature range, CaCl_2_ does not affect the packing of the DODAB vesicle core but increases the packing of the DODAB+GD3 vesicle core. EPR data also show that CaCl_2_ decreases the rigidity of both types of vesicles at temperatures above the gel–fluid transition. These results might help overcome the challenges of vaccine and immunotherapy cold chain logistics.

## Graphical abstract

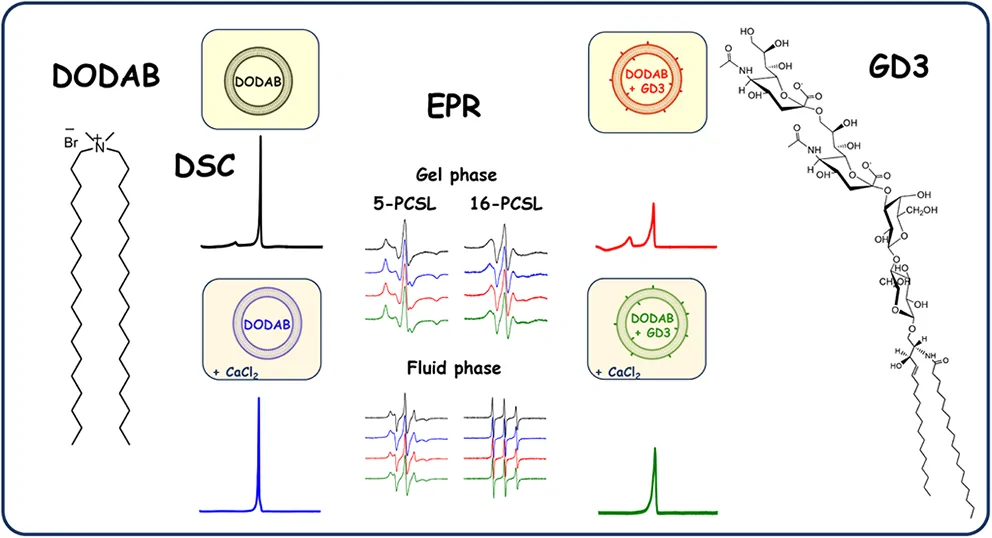

## 1 Introduction

The disialoganglioside GD3 is the precursor of the “b” series gangliosides,^1^ being the most abundant ganglioside in human colostrum.^2^ In mice, it is important for early neurological development, being abundant in neural stem cells.^3^ GD3 is involved with apoptosis control^4^ and is overexpressed in several types of cancer, such as melanoma, glioblastoma, and neuroblastoma.^5^ On the other hand, it has a low and restricted expression in extra-neural adult tissues.^6^ Moreover, it has been shown to induce responses by iNKT cells.^7,8^ This makes GD3 an attractive target for immunotherapy, and it has been tested in several clinical tests.^6^ The success of these tests might depend on the use of adequate adjuvants.

Dioctadecyldimethylammonium bromide (DODAB) is a synthetic cationic lipid that induces strong immune responses.^9^ DODAB vesicles have been successfully employed in drug delivery^10,11^ and as vaccine adjuvants.^9,12,13^ This makes DODAB a suitable carrier for GD3, and recently, DODAB vesicles containing GD3 had their structure characterized.^14^

One of the challenges in the development of new immunotherapeutic tools, such as vaccines, is the dependence on cold chain logistics, since freezing and thawing might affect their structures and stabilities.^15,16^ Indeed, there has been much effort in the development of protective excipients to stabilize vaccines.^16^

CaCl_2_ has been used in the formulation and stabilization of vaccines^17,18^ and in the preparation of drug delivery systems.^19^ The ionic pair that forms this salt also has strong biological relevance. For instance, Ca^2+^ ions are important second messengers whose concentrations are strictly controlled in the cellular milieu.^20^ They are also involved in important processes such as membrane fusion^21^ and the formation of membrane domains.^22^ Indeed, Ca^2+^ ions can interact with gangliosides and induce the lateral phase separation necessary to form the domains.^22–24^ Cl^–^ ions are also important to regulate cell cycle, proliferation, volume, and pH levels.^25^

The effects of CaCl_2_ on phospholipid membrane structures have been extensively studied^26^ and even employed in the preparation of drug and vaccine delivery systems, such as cochleates.^27^ However, the effects of CaCl_2_ on cationic vesicles have not been addressed thus far.

In the present work, the effects of CaCl_2_ on the structure of DODAB and DODAB+GD3 vesicles are characterized by differential scanning calorimetry (DSC) and electron paramagnetic resonance (EPR) spectroscopy. DSC gives information about the thermotropic behavior of lipid vesicles,^28^ and EPR spectroscopy gives information about lipid order and mobility at different depths of the assemblies.^29^ Indeed, DSC and EPR have been effectively combined to characterize mixtures of DODAB and other glycolipids.^30–32^

## 2 Experimental Methods

### 2.1 Materials

HEPES buffer (Lot# SLCB8158) and DODAB (Lot# BCBR1922 V) were purchased from Sigma Chemical Co. (St. Louis, MO, USA). CaCl_2_ was purchased from Honeywell/Fluka (Lot No. H265A). GD3 from bovine milk (Lot no. 860060–01–010) and spin labels 1-palmitoyl-2-(n-doxylstearoyl)-*sn*-glycero-3-phosphocholine (n-PCSL, n = 5 or 16) were purchased from Avanti Polar Lipids (Birmingham, AL, USA). Figure 1 shows the chemical structures of lipids and spin labels.

**Figure 1:**
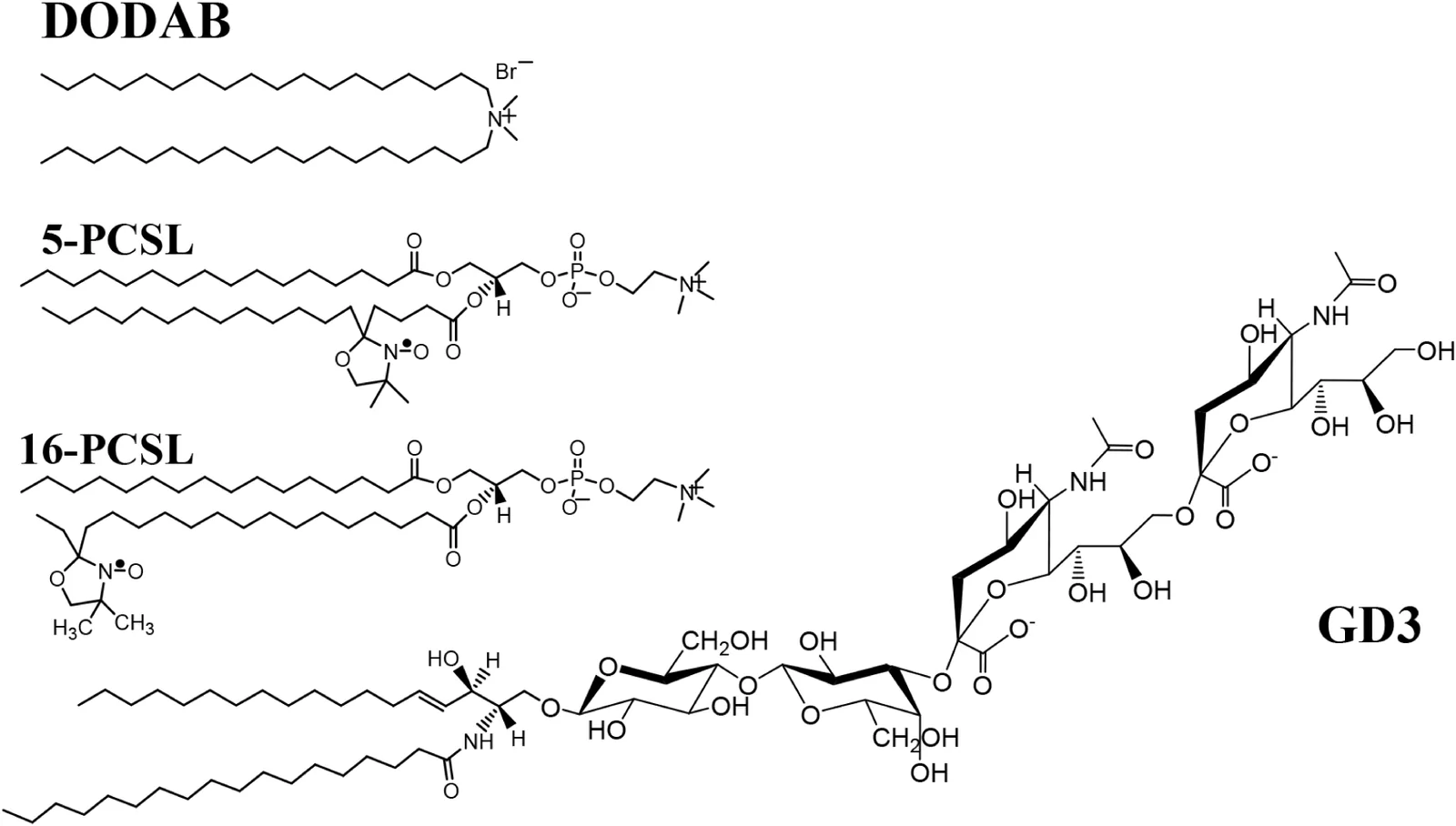
Chemical structures of DODAB, 5-PCSL, 16-PCSL, and GD3.

### 2.2 Preparation of Vesicles

Vesicles were prepared as previously described.^14^ Briefly, chloroform/methanol solutions (2:1 v/v) containing the lipids were dried under a nitrogen stream and left under reduced pressure for 2 h. The resulting lipid films were then dispersed in HEPES buffer (10 mM, pH 7.4) containing the desired CaCl_2_ concentration (0 mM, 1 mM, 10 mM, or 100 mM). The dispersion was achieved by heating the samples for 20 min at 87 °C in a water bath. Heating was interspersed with vortexing every 5 min to ensure a homogeneous dispersion.

For EPR experiments, 0.8 mol % of 5-PCSL or 0.3 mol % of 16-PCSL was added to the chloroform/methanol solutions before lipid film formation, and the samples were dispersed as described above. The final lipid concentrations were 2 mM DODAB and 0.1 mM GD3. At these concentrations, the preparations resulted in unilamellar vesicles.^14^

### 2.3 Differential Scanning Calorimetry (DSC)

DSC thermograms were obtained in a VP-DSC Microcalorimeter (Microcal Inc., Northampton, MA). Heating and cooling rates were set at 20 °C/h, and a heating (annealing) scan at 90 °C/h was performed before all experiments. Vesicles were equilibrated at 5 °C for 15 min before the heating scans. Scans were performed with at least two samples prepared on different days. Thermograms were analyzed using the Microcal Origin software, and the following thermodynamic parameters were determined: the phase transition temperature (T_m_), phase transition enthalpy (ΔH), and width at half-maximum (ΔT1/2). T_m_ is the highest value of heat capacity variation (ΔC_p_).^33^ ΔH is the enthalpy change per mol of lipid, and its values were obtained by integrating the area under the thermograms.^33^ ΔT_1/2_ values were obtained from the most intense peaks and reflect the phase transition cooperativity: the less cooperative a transition is, the larger the Δ*T*_1/2_ needed to complete it.^33^ All experiments were performed at least in duplicate with samples prepared on different occasions.

### 2.4 Electron Paramagnetic Resonance (EPR) Spectroscopy

In this work, the spin labels 5-PCSL and 16-PCSL were used to probe the regions near the surface and the core of the vesicles’ bilayers (Figure 1). These spin labels contain a nitroxide radical whose hyperfine structure consists of 3 lines. These lines have equal heights if the nitroxide group is moving rapidly in the EPR time scale. An increase in the immobilization of the spin label leads to differential line broadening in its EPR spectrum, and this anisotropy can be used to examine the environment of the probe. A detailed discussion of the EPR technique regarding its use to investigate the structure of cationic vesicles is given in ref 29.

A Bruker EMX spectrometer equipped with the ER4119HS high-sensitivity cavity was used to obtain CW-EPR spectra in the X-band. Sample temperatures were kept within 0.1 °C using a Bruker BVT-2000 variable temperature device. The values of maximum (A_max_) and minimum (A_min_) hyperfine splittings, and of low (h_+1_), central (h_0_) and high (h_‑1_) field amplitudes, were measured directly from the spectra (see Figures 3 and 5).

The effective order parameter, S_eff_, was calculated from the expression^34^

$$S_{eff}=\frac{A_{∥}-A_{⊥}}{A_{zz}-\left(1/2\right)\left(A_{xx}+A_{yy}\right)}\frac{a_{o}^{\prime }}{a_{o}}$$

where theoretical and experimental isotropic hyperfine splittings, $a_{o}^{\prime }$ and a_*o*_ respectively, can be calculated from the expressions: $a_{o}^{\prime }=\left(1/3\right)\left(A_{xx}+A_{yy}+A_{zz}\right)$ , a_o_ = (1/3)­(*A*_//_+2*A*_⊥_), A_//_ (= A_max_) is the maximum hyperfine splitting directly measured in the spectrum (see Figure 5), $A_{⊥}=A_{\min}+1.4\left[1-\frac{A_{//}-A_{\min}}{A_{zz}-\left(1/2\right)\left(A_{xx}+A_{yy}\right)}\right]$ , A_min_ is the measured inner hyperfine splitting (see Figure 5) and A_*xx*_, A*_yy_,* and A_*zz*_ are the principal values of the hyperfine tensor for doxylpropane.^35^

Each experiment was performed at least twice with fresh samples, and the standard deviations are shown as error bars.

## 3 Results

### 3.1 Effect of CaCl_2_ on the DODAB and DODAB+GD3 Vesicles Phase Behavior

The heating and cooling thermograms of pure DODAB vesicles and mixed DODAB + GD3 vesicles in the presence or absence of CaCl_2_ are shown in Figure 2.

**Figure 2:**
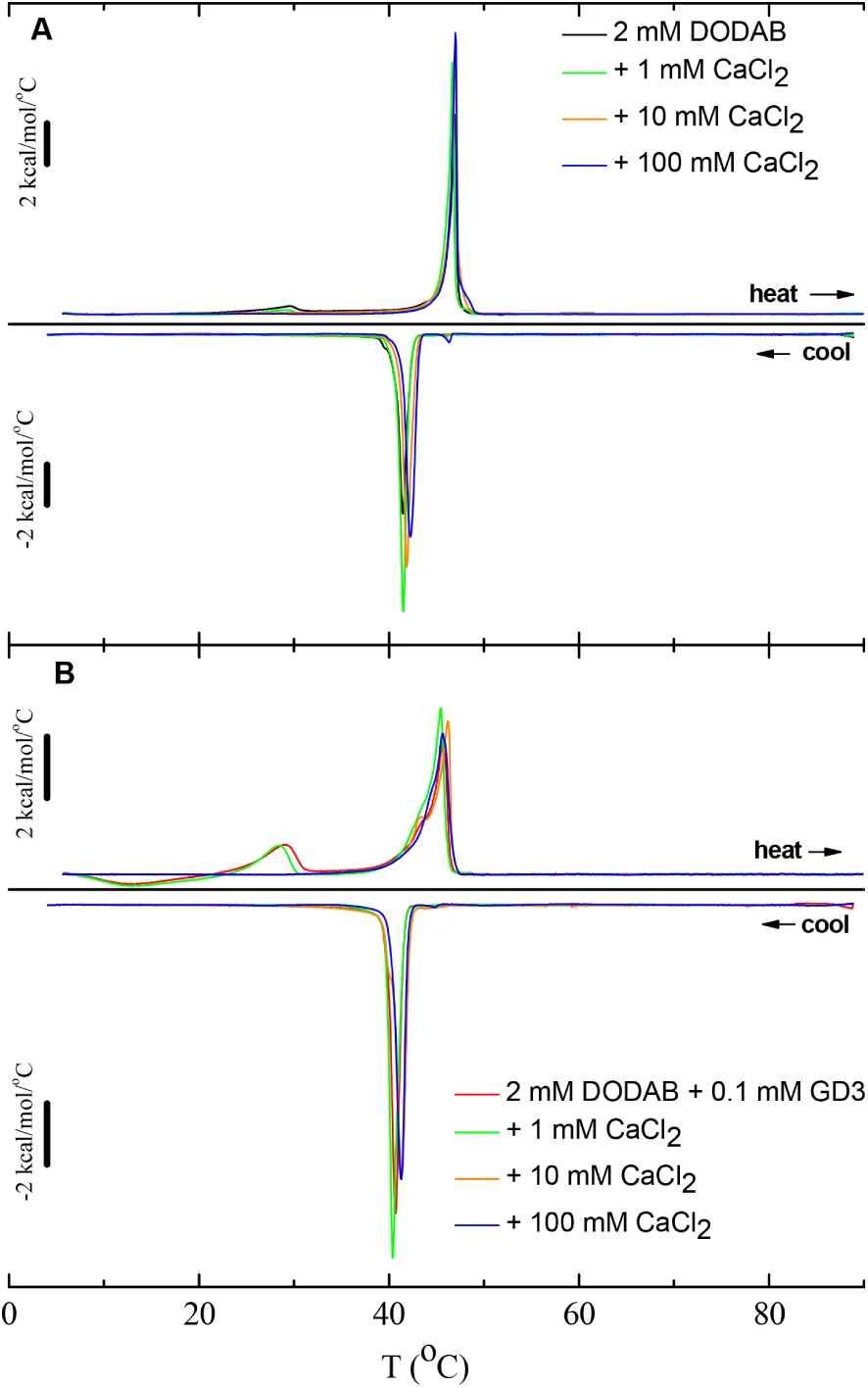
Effect of CaCl_2_ on the DSC thermograms of DODAB (A) and DODAB+GD3 (B) vesicles. Scan rates for heating and cooling were 20 °C/hour.

When heated, pure DODAB vesicles in the absence of CaCl_2_ display a small and broad endothermic peak at 29.9 °C, corresponding to the subgel-gel pretransition, and a sharp endothermic peak at 46.9 °C, corresponding to the main gel–fluid phase transition of unilamellar vesicles (Figure 2A). These peaks have been extensively described.^36,37^ In the presence of 1 mM CaCl_2_, a less intense subgel-gel peak is still observed, but in the presence of 10 mM and 100 mM CaCl_2_, this pretransition peak disappears (Figure 2A). The main gel–fluid peak remains nearly unchanged at 46.9 °C in the presence of all CaCl_2_ concentrations tested (Figure 2A, Table 1).

**Table 1:** Heating Cycle Thermodynamic Parameters (± Standard Deviations) of 2 mM DODAB and 2 mM DODAB + 0.1 mM GD3 Dispersions in the Absence and Presence of CaCl_2_

		Temperature range			Temperature range			Temperature range		
		5–20 °C			20–35 °C			35–80 °C		
Dispersion	[CaCl_2_] (mM)	T_m_ (°C)	ΔH (kcal/mol)	ΔT_1/2_ (°C)	T_m_ (°C)	ΔH (kcal/mol)	ΔT_1/2_ (°C)	T_m_ (°C)	ΔH (kcal/mol)	ΔT_1/2_ (°C)
		Heating cycle								
DODAB	0	-	-	-	29.9 ± 0.3	3.4 ± 0.9	4 ± 1	46.9 ± 0.1	14 ± 2	0.7 ± 0.1
DODAB	1	-	-	-	29.3 ± 0.3	1.3 ± 0.8	2.9 ± 0.4	46.7 ± 0.1	14 ± 2	0.6 ± 0.1
DODAB	10	-	-	-	-	-	-	47.0 ± 0.1	15.0 ± 0.6	0.6 ± 0.1
DODAB	100	-	-	-	-	-	-	47.0 ± 0.1	14.3 ± 0.8	0.6 ± 0.1
+ GD3	0	12 ± 1	-2.4 ± 0.2	7 ± 1	29.3 ± 0.3	5.2 ± 0.6	4.1 ± 0.1	45.8 ± 0.1	12.5 ± 0.3	1.7 ± 0.1
+ GD3	1	12.9 ± 0.1	-2 ± 1	9 ± 1	28.5 ± 0.1	3.7 ± 0.9	3.3 ± 0.2	45.5 ± 0.1	15 ± 2	1.7 ± 0.1
+ GD3	10	-	-	-	-	-	-	46.2 ± 0.1	13.8 ± 0.8	1.6 ± 0.2
+ GD3	100	-	-	-	-	-	-	45.7 ± 0.1	15 ± 1	2.2 ± 0.1

When cooled, the pure DODAB vesicles display a single sharp exothermic peak at 41.5 °C, corresponding to the fluid-gel transition (Figure 2A), as previously described.^32^ In the presence of 1 mM CaCl_2_ the peak remains at 41.5 °C, but in the presence of 10 mM and 100 mM CaCl_2_ it is shifted to somewhat higher temperatures (Figure 2A, Table 2).

**Table 2:** Cooling Cycle Thermodynamic Parameters (± Standard Deviations) of 2 mM DODAB and 2 mM DODAB + 0.1 mM GD3 Dispersions in the Absence and Presence of CaCl_2_

		Temperature range			Temperature range			Temperature range		
		5–20 °C			20–35 °C			35–80 °C		
Dispersion	[CaCl_2_] (mM)	*T*_m_ (°C)	ΔH (kcal/mol)	Δ*T*_1/2_ (°C)	*T*_m_ (°C)	ΔH (kcal/mol)	Δ*T*_1/2_ (°C)	*T*_m_ (°C)	ΔH (kcal/mol)	Δ*T*_1/2_ (°C)
		Heating cycle								
DODAB	0	-	-	-	-	-	-	41.5 ± 0.1	-11.7 ± 0.7	0.8 ± 0.1
DODAB	1	-	-	-	-	-	-	41.5 ± 0.1	-13 ± 2	0.7 ± 0.1
DODAB	10	-	-	-	-	-	-	41.8 ± 0.1	-14 ± 1	0.9 ± 0.1
DODAB	100	-	-	-	-	-	-	42.2 ± 0.1	-14 ± 2	1.0 ± 0.1
+ GD3	0	-	-	-	-	-	-	40.6 ± 0.1	-11.3 ± 0.4	1.3 ± 0.6
+ GD3	1	-	-	-	-	-	-	40.4 ± 0.1	-13.3 ± 0.1	1.0 ± 0.1
+ GD3	10	-	-	-	-	-	-	41.2 ± 0.2	-13.5 ± 0.7	1.1 ± 0.1
+ GD3	100	-	-	-	-	-	-	42.3 ± 0.1	-12 ± 2	1.0 ± 0.1

When heated, the mixed DODAB + GD3 vesicles display a broad and shallow exothermic peak around 12 °C, followed by a more intense endothermic subgel-gel pretransition peak at 29.3 °C (Figure 2B). An endothermic peak at 45.8 °C corresponds to the main gel–fluid transition (Figure 2B); it is broader and less intense than the one observed for pure DODAB vesicles (Figure 2A). This complex thermotropic behavior upon heating has already been described and discussed for mixed DODAB+GD3 vesicles.^14^

The DODAB + GD3 heating thermogram profile is not significantly changed in the presence of 1 mM CaCl_2_, except that the endothermic pretransition peak is shifted to 28.5 °C (Figure 2B). In the presence of 10 mM and 100 mM CaCl_2_, the exothermic peak around 12 °C and the endothermic pretransition peak disappear (Figure 2B). With these higher CaCl_2_ concentrations, the endothermic peak corresponding to the main gel–fluid transition remains around 45.8 °C (Figure 2B).

When cooled, the mixed DODAB + GD3 vesicles have a single sharp exothermic peak at 40.6 °C, corresponding to the fluid-gel transition (Figure 2B).^14^ This peak shifts to higher temperatures in the presence of CaCl_2_ concentrations above 1 mM (Figure 2, Table 2).

As observed in Figure 2, the heating and cooling cycles result in very different thermogram profiles for both pure and mixed vesicles. Hence, the thermodynamic parameters obtained from these thermograms are summarized in Table 1 for the heating cycles and in Table 2 for the cooling cycles.

The thermodynamic analyses were performed in separate temperature ranges to facilitate the discussion of the thermal events observed in the thermograms. A similar approach was previously used for the analyses of mixed DODAB + GD3 vesicles.^14^ For instance, the temperature range of 5 to 20 °C is suitable for the analysis of the exothermic peak at 12 °C that appears in the heating scans of mixed DODAB + GD3 vesicles (Figure 2B). The T_m_, ΔH, and ΔT_1/2_ values for this peak are not significantly affected by the presence of 1 mM CaCl_2_ (Table 1). However, in the presence of higher CaCl_2_ concentrations, this peak is absent (Figure 2B).

In the temperature range of 20 to 35 °C, it is possible to observe that 1 mM CaCl_2_ causes a decrease in the pretransition ΔH for both pure DODAB and mixed DODAB + GD3 vesicles (Table 1). This concentration also causes a slight increase in pretransition cooperativity for both kinds of vesicles, since ΔT_1/2_ values are decreased in the presence of 1 mM CaCl_2_ (Table 1). In mixed vesicles, 1 mM causes a shift in the position of the pretransition peak from 29.3 to 28.5 °C (Table 1), as observed in Figure 2B. In the presence of higher CaCl_2_ concentrations, there is no pretransition peak (Figure 2A and B).

In the temperature range of 35 to 90 °C, increasing CaCl_2_ concentrations does not significantly affect the T_m_, ΔH, and ΔT_1/2_ values of the gel–fluid transition for pure DODAB vesicles (Table 1). For mixed DODAB + GD3 vesicles, the increase in CaCl_2_ concentrations does not significantly affect the T_m_ values but causes a slight increase in ΔH and ΔT_1/2_ values (Table 1). This effect of CaCl_2_ concentration on ΔH and ΔT_1/2_ is more evident in the presence of 100 mM CaCl_2_ (Table 1).

For the cooling cycles (Table 2), it is possible to observe that CaCl_2_ does not affect the ΔH and ΔT_1/2_ values for pure DODAB vesicles, but it increases the T_m_ values for CaCl_2_ concentrations above 1 mM. A similar behavior is observed for mixed DODAB+GD3 vesicles is the presence of CaCl_2_ (Table 2).

For both pure DODAB and mixed DODAB + GD3 vesicles, the gel–fluid and fluid–gel transitions do not occur at the same temperature (Figure 2, Tables 1 and 2). This temperature hysteresis can be quantified as the difference between T_m_ values for heating and cooling cycles, as shown in Table 3.

**Table 3:** Temperature Hysteresis (± Standard Deviations) between Heating and Cooling Cycles for 2 mM DODAB and 2 mM DODAB + 0.1 mM GD3 Dispersions in the Absence and Presence of CaCl_2_

Dispersion	[CaCl_2_] (mM)	T_m_ heating (°C)	T_m_ cooling (°C)	Hysteresis (Tm heating – Tm cooling) (°C)
DODAB	0	46.9 ± 0.1	41.5 ± 0.1	5.4 ± 0.2
DODAB	1	46.7 ± 0.1	41.5 ± 0.1	5.2 ± 0.2
DODAB	10	47.0 ± 0.1	41.8 ± 0.1	5.2 ± 0.2
DODAB	100	47.0 ± 0.1	42.2 ± 0.1	4.8 ± 0.2
+ GD3	0	45.8 ± 0.1	40.6 ± 0.1	5.2 ± 0.2
+ GD3	1	45.5 ± 0.1	40.4 ± 0.1	5.1 ± 0.2
+ GD3	10	46.2 ± 0.1	41.2 ± 0.2	5.0 ± 0.3
+ GD3	100	45.7 ± 0.1	42.3 ± 0.1	3.4 ± 0.2

Pure DODAB vesicles, in the absence of CaCl_2_ have a thermal hysteresis of 5.4 °C, as previously described.^36,38^ For these vesicles, a slight decrease in hysteresis is observed as CaCl_2_ concentrations are increased (Table 3). Mixed DODAB + GD3 vesicles have a hysteresis of 5.2 °C, very similar to that of pure DODAB vesicles, as previously described.^14^ For mixed vesicles, the hysteresis also decreases as CaCl_2_ concentration increases, and this effect is more evident in the presence of 100 mM CaCl_2_, since hysteresis is reduced by almost 2 °C (Table 3).

Considering that the thermodynamic differences observed in Figure 2 and Tables 1, 2, and 3 are more evident in the presence of 100 mM CaCl_2_, this saturating concentration was chosen to investigate the effect of CaCl_2_ on the structure of the vesicles by EPR spectroscopy.

### 3.2 Effect of CaCl_2_ on the Structure of DODAB and DODAB + GD3 Vesicles at Temperatures below the Gel–Fluid Transition

The structures of DODAB or DODAB + GD3 vesicles, in the presence and absence of 100 mM CaCl_2_ were compared using the paramagnetic probes 5-PCSL and 16-PCSL (Figure 1). These probes give information about the microviscosity and packing near the interface and the core of lipid bilayers, respectively.^29^ The very small concentrations of probes used in this work do not affect the structure or thermotropic behavior of the vesicles. Indeed, the presence of these probes does not affect the thermograms of the vesicles (see Supporting Information Figure 1).

Figure 3 shows the spectra of these probes embedded in vesicles in the presence or absence of CaCl_2_ at temperatures below the gel–fluid transition. For clarity, spectra at other temperatures in this range are shown in Supporting Information Figure 2.

**Figure 3:**
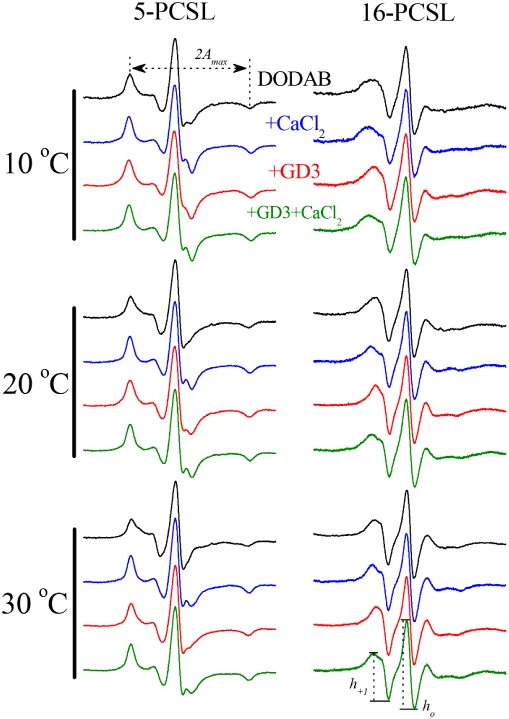
EPR spectra of 5-PCSL and 16-PCSL embedded in 2 mM DODAB vesicles or in 2 mM DODAB + 0.1 mM GD3 vesicles, with or without 100 mM CaCl_2_, at temperatures below the gel–fluid transition. The maximum hyperfine splitting (A_max_) and the amplitudes of low (h_+1_) and central (h_0_) field lines are indicated. The total spectra width is 100 G.

The spectra of 5-PCSL and 16-PCSL are very different at all temperatures and for all samples tested (Figure 3). While the spectra of 5-PCSL are characteristic of an anisotropic environment, in which the movement of the probe is more restricted due to increased molecular packing, the spectra of 16-PCSL are typical of a more isotropic environment, where the probe has higher mobility and lower order due to decreased molecular packing (Figure 3).^35^

Hence, the spectra of 5-PCSL and 16-PCSL show the presence of a gradient of increased flexibility from the surface to the core of the bilayers, which is typical of noninterdigitated lamellar structures.^35^ This flexibility gradient has been described for DODAB vesicles in the gel phase,^29,31^ and from Figure 3 it is possible to observe that it is not affected by the presence of GD3, CaCl_2_, or both.

The spectra display features of a single EPR signal at all conditions tested (Figure 3, Supplementary Figure 2). Considering that both probes are miscible with DODAB and GD3,^14,39^ the presence of a single EPR signal shows that these lipids are miscible and that no lateral phase separation is induced by CaCl_2_ at these temperatures. The presence of a single EPR signal allows for the determination of empirical parameters from the spectra. These parameters can be used to compare the structures of vesicles under different conditions.^29^

The maximum hyperfine splitting (A_max_), for instance, can be directly obtained from the 5-PCSL spectra (Figure 3). A_max_ is sensitive to the packing and viscosity of the probe microenvironment: a decrease in packing or viscosity results in smaller A_max_ values.^29^ In contrast, A_max_ values cannot be accurately determined from 16-PCSL spectra due to increased isotropy (Figure 3). For this probe, structural information can be obtained from the ratio of the low- and central-field-line amplitudes (h_+1_/h_0_) (Figure 3). The values of the h_+1_/h0 ratio increase as the bilayers become less packed.^29^

Figure 4 shows the *A*_*max*_ and h_+1_/h_0_ values for DODAB and DODAB+GD3 vesicles in the presence and absence of CaCl2 at temperatures below the gel–fluid transition.

**Figure 4:**
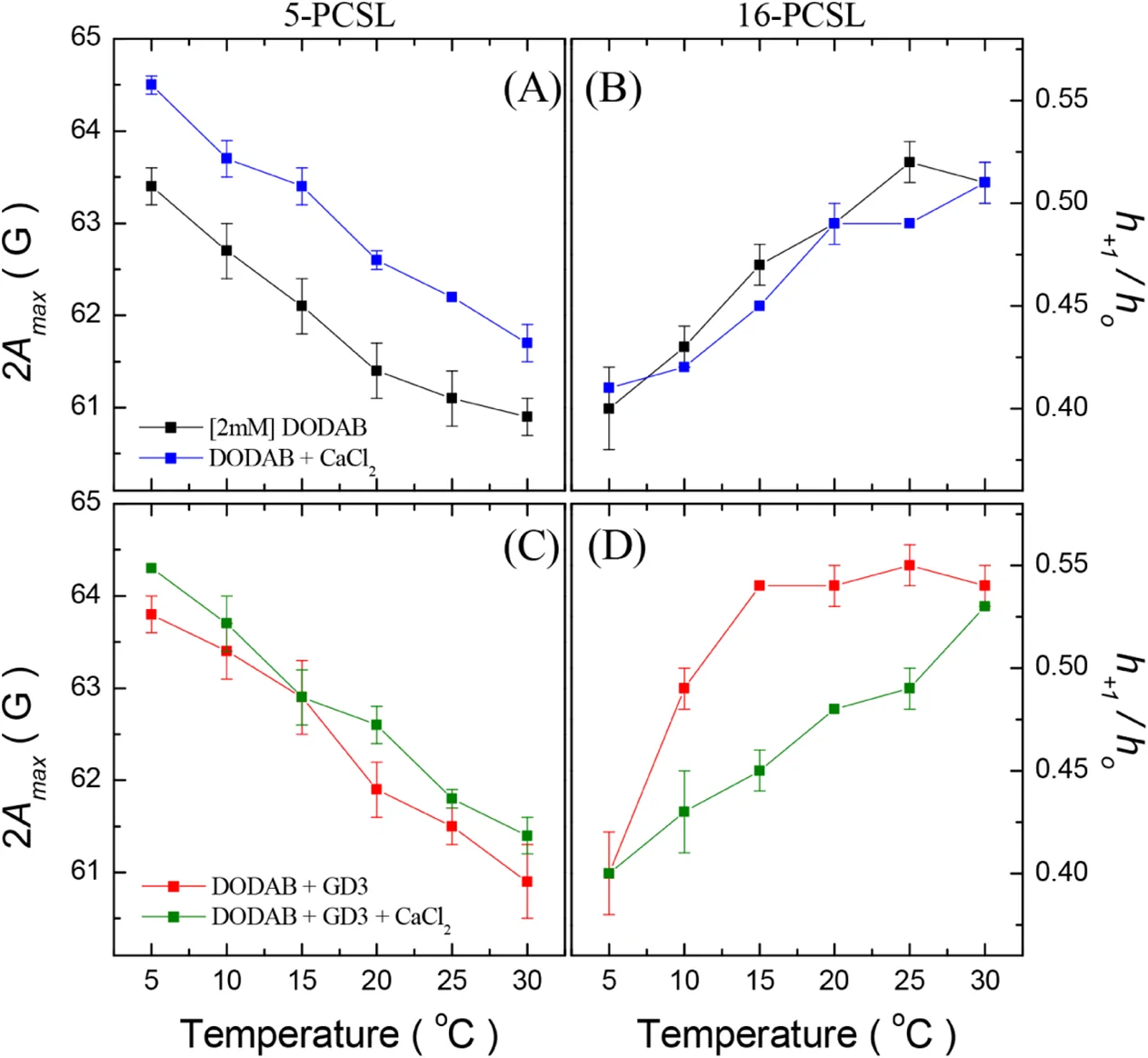
Effect of 100 mM CaCl_2_ on the maximum hyperfine splitting (A_max_) of 5-PCSL (A, C) and on the ratio of the low and central field line amplitudes (h_+1_/h_0_) of 16-PCSL (B, D) embedded in 2 mM DODAB or 2 mM DODAB + 0.1 mM GD3 vesicles at temperatures below the gel–fluid transition. Error bars indicate standard deviations.

As expected, A_max_ values decrease as the temperature increases because membrane packing decreases as temperature increases (Figure 4A,C). In the presence of CaCl_2_, the A_max_ values for pure DODAB vesicles increase, indicating that CaCl_2_ increases the superficial packing of these vesicles (Figure 4A). A similar but less intense effect is observed for DODAB+GD3 vesicles (Figure 4C).

The decrease in packing with temperature increase is also observed in the membrane core, since h_+1_/h_0_ values increase (Figure 4B,D). For pure DODAB vesicles, CaCl_2_ does not have a clear effect on membrane core packing (Figure 4B). For DODAB+GD3 vesicles, h_+1_/h_0_ values decrease in the presence of CaCl_2_, indicating that CaCl_2_ induces an increase in DODAB+GD3 membrane core packing. Hence, CaCl_2_ has a rigidifying effect on vesicles formed by a mixture of DODAB and GD3 at temperatures below the gel–fluid transition.

### 3.3 Effect of CaCl_2_ on the Structure of DODAB and DODAB + GD3 Vesicles at Temperatures above the Gel–Fluid Transition

The spectra of 5-PCSL and 16-PCSL embedded in vesicles, in the presence or absence of CaCl_2_ at temperatures above the gel–fluid transition, are shown in Figure 5.

**Figure 5:**
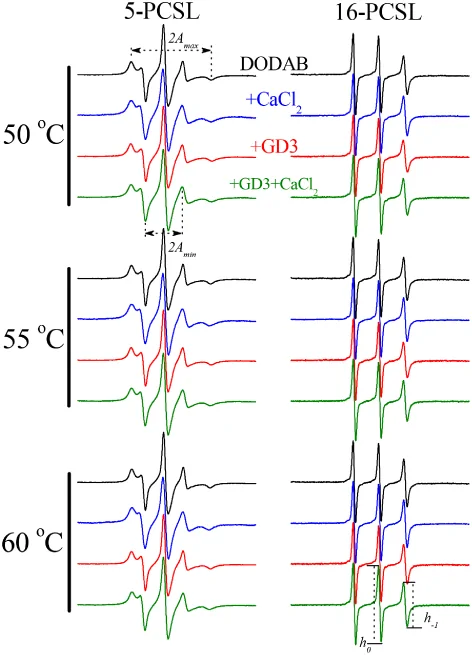
EPR spectra of 5-PCSL and 16-PCSL embedded in 2 mM DODAB vesicles or in 2 mM DODAB + 0.1 mM GD3 vesicles, with or without 100 mM CaCl_2_, at temperatures above the gel–fluid transition. The maximum and minimum hyperfine splittings (A_max_ and A_min_), and the amplitudes of central (h_0_) and high (h_‑1_) field lines are indicated. The total spectral width is 100 G.

When compared to the spectra at temperatures below the gel–fluid transition (Figure 3), the spectra of 5-PCSL shown in Figure 5 have thinner features, which indicate that the movement down the long axis of this probe is faster. This is typical of a fluid yet microscopically ordered structure close to the membrane interface.^35^ Similarly, the spectra of 16-PCSL at higher temperatures display sharp peaks (Figure 5), indicating a fast and nearly isotropic movement of the nitroxide label, which is typical of the fast motional regime observed in fluid bilayers.^35^

The spectra of both probes are very similar under all conditions tested (Figure 5), so the empirical parameters can be more informative about the structural organization of DODAB and DODAB+GD3 vesicles in the presence and absence of CaCl_2_ above the gel–fluid transition.

The maximum and minimum hyperfine splittings (A_max_ and A_min_) can be accurately measured in the 5-PCSL spectra (Figure 5). With their values, it is possible to calculate the effective order parameter (S_eff_, see Section 2.4), which gives information about the hydrocarbon chain order.^40,41^

The 16-PCSL spectra sense a less ordered environment, so the hyperfine splittings are not clearly defined (Figure 5). However, the amplitudes of the high (h_‑1_) and central field lines (h_0_) can be accurately measured (Figure 5). The ratio of these amplitudes (h_‑1_/h_0_) can be used to assess the viscosity and packing in the same manner as described for *h*_*+1*_/*h*_0_ in the previous section: the values of h_‑1_/h_0_ increase as the membrane viscosity decreases.^29^

The values of S_eff_ and h_‑1_/h_0_ for DODAB and DODAB+GD3 vesicles, in the presence and absence of CaCl_2_ at temperatures above the gel–fluid transition, are shown in Figure 6.

**Figure 6:**
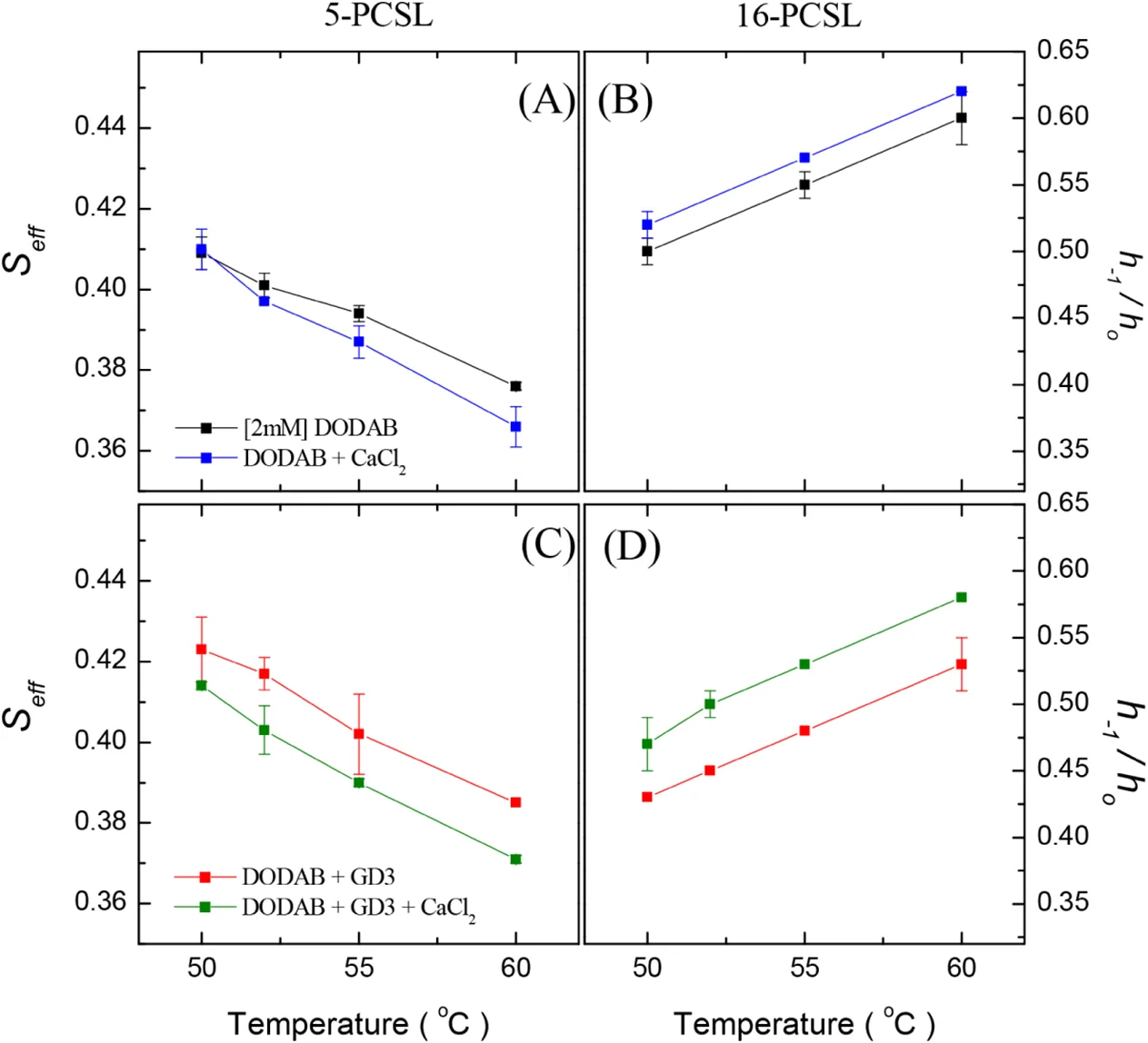
Effect of 100 mM CaCl_2_ on the effective order parameter (S_eff_) of 5-PCSL (A, C) and on the ratio of the central and high field line amplitudes (h_‑1_ */*h_0_) of 16-PCSL (B, D) embedded in 2 mM DODAB or 2 mM DODAB + 0.1 mM GD3 vesicles at temperatures above the gel–fluid transition. Error bars indicate standard deviations.

S_eff_ values are smaller in the presence of CaCl_2_ for both DODAB and DODAB+GD3 vesicles (Figure 6A,C), showing that CaCl_2_ decreases the superficial order of these vesicles. Similarly, CaCl_2_ decreases the molecular packing of the membrane core of both DODAB and DODAB+GD3 vesicles, as seen from the higher h_‑1_/h_0_ values in the presence of this salt (Figure 6B,D). In summary, CaCl_2_ decreases the rigidity of DODAB and DODAB+GD3 vesicles in the fluid phase.

## 4 Discussion

Pure DODAB vesicles have a rich phase behavior, which depends on temperature, concentration, and the presence of additives.^42^ In the diluted regime tested here, DODAB vesicles can form subgel, gel, and fluid phases.^42,43^

The subgel phase is formed when the vesicles are cooled below 15 °C.^36,37^ This cooling enables a reorganization of DODAB molecules from a close-to-hexagonal to a close-to-triclinic 2D lattice.^37,38^ This new molecular arrangement results in more tightly packed acyl chains, with a consequent freezing of the chains’ motion and their further stretching.^37,38^

When subgel phase vesicles are heated, a pretransition peak appears on the thermograms as a reflex of the energy needed to complete the order–disorder transition to the gel phase (Figure 2A). For pure DODAB vesicles prepared in water, the pretransition peak is centered at 36 °C and is almost as intense as the main gel–fluid transition peak.^37,38^ For vesicles prepared in HEPES buffer, the pretransition peak is shifted to 29.9 °C, and the peak is significantly decreased when compared to the gel–fluid transition peak, as observed in Figure 2A and Table 1, and as previously described.^14^ These differences suggest that the increase in ionic strength might affect the formation of the subgel phase due to electrostatic shielding. Indeed, the pretransition peak decreases and disappears as CaCl_2_ concentration increases in the buffered samples (Figure 2A, Table 1).

Although the chloride ion (Cl^–^) has a smaller polarizability and binding affinity to the quaternary ammonium headgroup when compared to bromide ions (Br^–^),^44–46^ at saturating concentrations, it might effectively shield the electrostatic repulsion between cationic DODAB headgroups and could inhibit the transformation of a gel-phase hexagonal arrangement to a subgel triclinic arrangement. In agreement with that, CaCl_2_ does not significantly affect the acyl chain packing at the membrane core (Figure 4B), but it does increase the superficial packing of the vesicles (Figure 4A). Similarly, the cationic lipid didodecyldimethylammonium bromide (DDAB) displays a phase-separated region with two coexisting lamellar phases.^47,48^ One of these phases disappears when Br^–^ ions are replaced by Cl^–^ ions.^48,49^

The mixed DODAB+GD3 vesicles also display a pretransition peaking at 29.3 °C when heated, which is preceded by a broad and shallow exothermic peak around 12 °C (Figure 2B, Table 1). This suggests that GD3 does not interfere with the formation of the subgel phase, but that this subgel phase is possibly metastable, with a superficial condensation effect caused by the bulky and charged headgroup and a decrease in packing of the core due to steric effects of the hydrophobic chains of GD3.^14^ Stepwise changes that reflect head/tail differences in molecular interactions have already been described for DODAB during the fluid-to-coagel phase transition.^50^

Both exothermic and pretransition peaks disappear as CaCl_2_ concentrations increase (Figure 2B, Table 1). The disappearance of these peaks is probably related to the electrostatic shielding effects of CaCl_2_. As discussed above, Cl^–^ shielding of cationic headgroups could inhibit the rearrangement of DODAB molecules into a subgel phase. Moreover, Ca^2+^ could interact with the sialic acid residues of GD3 (Figure 1), changing their conformation and transmitting this conformational change to the GD3 hydrocarbon chains. It was shown that CaCl_2_ induces a micellar-to-lamellar change in dispersions of pure GD3, with an increase in superficial and core rigidity.^39^ Although the vesicles are already arranged as lamellae, a similar effect is observed here (Figure 4): in the presence of CaCl_2_ there is a superficial packing increase (Figure 4C) and an increase in packing of the membrane core (Figure 4D).

After the pretransition is completed, pure DODAB vesicles are at gel phase, and a single peak at 46.9 °C corresponding to the gel–fluid transition is observed (Figure 2A and Table 1). This transition temperature is higher than the one at 44 °C described for DODAB vesicles prepared in water,^36–38^ probably because the HEPES buffer has an electrostatic shielding effect that increases the molecular packing of the gel phase.

CaCl_2_ does not significantly affect the T_m_, ΔH, and ΔT_1/2_ values of the gel–fluid transition for pure DODAB vesicles at any of the concentrations tested (Table 1), but a substantial increase in superficial packing is observed in the presence of 100 mM CaCl_2_ (Figure 4A). On the other hand, CaCl_2_ does not affect the molecular packing of the membrane core (Figure 4B). This suggests that although Cl^–^ ions can shield the electrostatic repulsion between DODAB headgroups and increase their packing, the Cl^–^ ions do not affect the overall molecular packing and thermotropic behavior of a gel phase that is already more packed due to HEPES buffer when compared to pure water, as discussed above.

In the mixed DODAB+GD3 vesicles, the gel–fluid phase transition temperature and cooperativity are decreased when compared to pure vesicles (Figure 2B,A, Table 1). This could be explained by a condensation effect of the bulky and charged GD3 headgroup, that increases the superficial rigidity of DODAB vesicles, combined with a decrease in membrane core rigidity caused by the V-shaped configuration of GD3 ceramide that occupies a large area and disrupts hydrocarbon chain interactions of DODAB molecules, as previously described.^14^

CaCl_2_ does not affect the T_m_ of the mixed vesicles, and a slight increase in ΔH and ΔT_1/2_ values is only observed in the presence of 100 mM CaCl_2_ (Figure 2B, Table 1). The increase in ΔH values could be explained by the rigidifying effect of CaCl_2_ on the surface and core of the mixed vesicles (Figure 4C and D). This rigidifying effect could be due to conformational changes in the headgroups and hydrocarbon chains induced by Ca^2+^ interactions with the sialic acid residues of GD3 (Figure 1), as discussed before.

The increase in ΔT_1/2_ values indicates that 100 mM CaCl_2_ decreases the gel–fluid transition cooperativity in mixed vesicles (Table 1). This result is quite unexpected, since the increase in ΔH (Table 1) and the rigidity of both the membrane surface and core (Figure 4C and D) would point instead to an increase in cooperativity. It is known that cooperativity does not correlate well with other thermodynamic parameters because it is sensitive to the specific character of intermolecular chain packing, such that the ability to communicate chain disordering can be very inefficient in either very tight or very weak packing lattices.^51^ Indeed, cooperativity is dependent on the morphology of bilayers,^52^ and it was shown that DODAB bicelles prepared by sonication present a sharp decrease in cooperativity when compared to DODAB vesicles.^53,54^ These bicelles exhibit EPR spectra with two signals corresponding to the coexistence of a gel planar domain and a fluid rim domain.^29^ Hence, this decrease in cooperativity suggests that 100 mM CaCl_2_ could be changing the morphology of mixed DODAB+GD3 vesicles, but this possible change does not seem to be so drastic as to induce the formation of bicelles, because the EPR spectra shown in Figure 3 and Supplementary Figure 2 display a single signal. The formation of curled or faceted vesicles is more likely.^30,38^

In the cooling cycles, concentrations of CaCl_2_ above 1 mM induce an increase in the fluid-gel transition temperature for pure DODAB vesicles (Table 2), indicating that it is not necessary to cool down the samples as much as in the absence of CaCl_2_ to transition to the gel phase. This increase in the T_m_ for cooling results in a decrease in hysteresis from 5.4 °C in the absence of CaCl_2_ to 4.8 °C in the presence of 100 mM CaCl_2_ (Table 3). Temperature hysteresis is common in charged lipids^55^ because the paths for gel–fluid and fluid-gel transitions are different: near the transition temperature, domains of one phase are present in the bulk matrix of the other phase, resulting in strain energy differences.^55^ If all these domains were collected into a single large domain, hysteresis would be reduced because the strain energy would also be reduced. Hence, the decrease in hysteresis caused by CaCl_2_ suggests that this salt can reduce strain energy, possibly by facilitating the formation of larger domains near the transition temperature.

The effect of different counterions on the structure of DODAB vesicles has been explored for the gel phase.^45,56^ It was shown that the smaller and less hydrated Br^–^ ion has a larger binding affinity for the quaternary ammonium headgroup than Cl^–^, as expected from the Hofmeister series effects.^45,46^ This means that Br^–^ as a counterion yields a “softer”, less tightly packed vesicle when compared to Cl^–^.^45^ The EPR data in Figure 6A and B, however, show that in the presence of CaCl_2_, the DODAB vesicles are less tightly packed than in absence of CaCl_2_. A possible explanation for this difference is that specific ion (Hofmeister series) effects are dependent on temperature.^57^

Similar effects of increasing fluid-gel transition temperature (Table 2), decreasing hysteresis (Table 3), and decreasing the fluid phase packing (Figure 6C and D), are observed for mixed DODAB+GD3 vesicles in the presence of CaCl_2_. In this case, the effects are stronger than for the pure DODAB vesicles (Tables 2 and 3, Figure 6C and D), suggesting that the interaction of Ca^2+^ ions with the sialic acid of GD3 headgroups might be playing a synergistic role with the Cl^–^ interacting with the DODAB headgroups.

GD3 is an attractive target for cancer immunotherapy,^58^ but clinical trials exploring this ganglioside did not achieve major impacts on patient outcomes so far.^6^ This could be due to the use of inadequate adjuvants, since cancer immunotherapy strongly depends on strong cellular responses.^59^ Since DODAB is able to induce the formation of both humoral and cellular responses,^9,12^ vesicles of DODAB containing GD3 had their structures recently characterized to enable the development of new immunotherapeutic tools.^14^

DODAB vesicles would be interesting as adjuvants for GD3-based therapies because cationic vesicles are known to be more potent adjuvants than neutral and anionic ones.^60^ Moreover, vesicles with higher gel–fluid transition temperatures usually elicit stronger immune responses,^60^ and Table 1 shows that all vesicles tested here have gel–fluid phase transitions well above the physiological temperature of 37 °C.

Temperature is also critical for effective vaccination programs because vaccines have an inherent thermal instability that requires a cold chain of storage and distribution.^15,61^ While mRNA-based vaccines are highly unstable when not frozen,^16^ most other types of vaccines rely on a cold chain with temperatures in the range of 2 to 8 °C.^15^ This is the case for nonreplicating vaccines, such as inactivated viruses/bacteria, purified subunit protein and carbohydrate antigens, virus-like particles, and vesicles that can be formulated as liquid solutions and stored in glass vials or prefilled syringes.^15^ These kinds of vaccines usually need stabilizers for the cold chain.^15,62,63^

The present work expands the characterization of DODAB+GD3 vesicles by exploring the effects of CaCl_2_, which has been used to stabilize vaccines.^18,19,64^ For the initial thermodynamic characterization using DSC, 1 mM, 10 mM, and 100 mM CaCl_2_ were tested (Figure 2 and Tables 1, 2 and 3). These concentrations were chosen because CaCl_2_ is a divalent salt: for instance, 1 mM CaCl_2_ would dissociate into 2 mM negative charges from Cl^–^ anions and 2 mM positive charges from Ca^2+^ cations. This would allow for the negative Cl^–^ charges to interact in an equimolar proportion to the monovalent cationic headgroup of 2 mM DODAB (Figures 1 and 2), and for the positive cationic Ca^2+^ charges to interact in a 10:1 proportion to the two anionic sialic residues of 0.1 mM GD3 (Figures 1 and 2).

It was shown that the stabilizing effects of CaCl_2_ are not linear with its concentration: for instance, foot-and-mouth disease vaccines have increased thermal stability with the addition of up to 10 mM CaCl_2_, while a decreasing trend was observed for concentrations above 30 mM CaCl_2_.^18^ Hence, it was important for this work to test higher concentrations. The initial concentration of 1 mM CaCl_2_ was increased by 10- and 100-fold, and the thermodynamic differences observed in Figure 2 and Tables 1, 2, and 3 are more evident in the presence of 100 mM CaCl_2_. Consequently, this saturating concentration was chosen to investigate the effect of CaCl_2_ on the structure of the vesicles by EPR spectroscopy, as previously mentioned.

As with other liquid vaccine formulations, the DODAB and DODAB+GD3 vesicles would most likely be kept in a 2 to 8 °C cold chain,^15^ which coincides with the temperature range of the subgel phase (Figure 2).^37,38^ The temperature of 5 °C to begin the DSC and EPR experiments, was chosen for this reason, and the disappearance of the subgel phase with increasing CaCl_2_ concentrations (Figure 2) could have impacts on vaccine distribution and/or potency.

The formation of the subgel phase creates a line tension with the gel phase, possibly because these phases have different thicknesses, which result in enhanced hydrophobic defects at the nucleating boundary.^37^ Since gel-phase DODAB vesicles are known to have many hydrophobic defects,^65,66^ which can be observed in the faceted aspect of gel-phase giant unilamellar vesicles,^30^ it is possible that these defects are further enhanced in the subgel phase. Since hydrophobic defects destabilize membranes and facilitate membrane fusion,^67^ it is possible that the subgel phase is more prone to fusion events than the gel phase. Hence, the disappearance of the subgel phase with the addition of CaCl_2_ might stabilize the DODAB vesicles at cold-chain temperatures.

Understanding the mechanisms of vaccine inactivation is very important to preserve their potency,^68,69^ just as understanding the structure, stability, and fluidity of vesicles is key for their use in vaccines.^60^ Hence, characterizing the effects of CaCl_2_ on the structure and the thermotropic phase behavior of DODAB and DODAB+GD3 vesicles is very important for their application in immunotherapy.

## 5 Conclusions

The effect of CaCl_2_ on the structure of DODAB and DODAB+GD3 vesicles was characterized by DSC and EPR spectroscopy. EPR spectra show that CaCl_2_ does not induce the formation of GD3 domains by lateral phase separation. DSC thermograms show that CaCl_2_ abolishes the DODAB subgel pretransition and the heating scan’s exothermic peak in vesicles containing GD3. It is possible that Cl^–^ ions hinder the transformation of a gel to a subgel phase by shielding the cationic headgroups’ electrostatic repulsion in vesicles cooled below 15 °C.

The thermograms also show that CaCl_2_ decreases the hysteresis between gel–fluid and fluid–gel transitions in both pure and mixed vesicles.

Empirical analysis of EPR spectra shows that CaCl_2_ increases the superficial rigidity of DODAB and DODAB+GD3 vesicles at temperatures below the gel–fluid transition. In this temperature range, CaCl_2_ does not affect the packing of the DODAB vesicle core but increases the packing of the core of DODAB+GD3 vesicles.

The empirical analysis also shows that CaCl_2_ decreases the superficial order and the molecular packing of the core of DODAB and DODAB+GD3 vesicles at temperatures above the gel–fluid transition.

Although the effects of CaCl_2_ on phospholipid membrane structures have been extensively studied, this is the first work to investigate its effects on cationic membranes containing gangliosides. The results shown here might help develop stable dispersions that can withstand the challenges of vaccine and immunotherapy cold-chain logistics.

## Supplementary Material



## Notes

Thermograms of vesicles containing spin labels and additional EPR spectra at temperatures below the gel–fluid transition (PDF)
