# Supplementary material for: Effect of CaCl2 on the Structure of DODAB Vesicles Containing the Ganglioside GD3
Source: ACS Omega. 2026 Aug 17;11(34):51191–201. doi: 10.1021/acsomega.6c03945 (PMC13625159; doi:10.1021/acsomega.6c03945)
Supplement: Supplementary file 1 [file ao6c03945_si_001.pdf]

Effect of $CaCl_2$ on the structure of DODAB vesicles containing the ganglioside GD3

Julia B. Ejarque[a], Evandro L. Duarte[b], M. Teresa Lamy[b] and Julio H. K. Rozenfeld[a,*]

[a]Departamento de Biofísica, Escola Paulista de Medicina, Universidade Federal de São Paulo, R. Botucatu 862, 04023-062, São Paulo, SP, Brazil

[b]Instituto de Física, Universidade de São Paulo, Rua do Matão 1371, 05508090, São Paulo, SP, Brazil

*Corresponding author. Phone number: +55 11 3385 4343 extension 2340. E-mail address: julio.rozenfeld@unifesp.br (J.H.K. Rozenfeld)

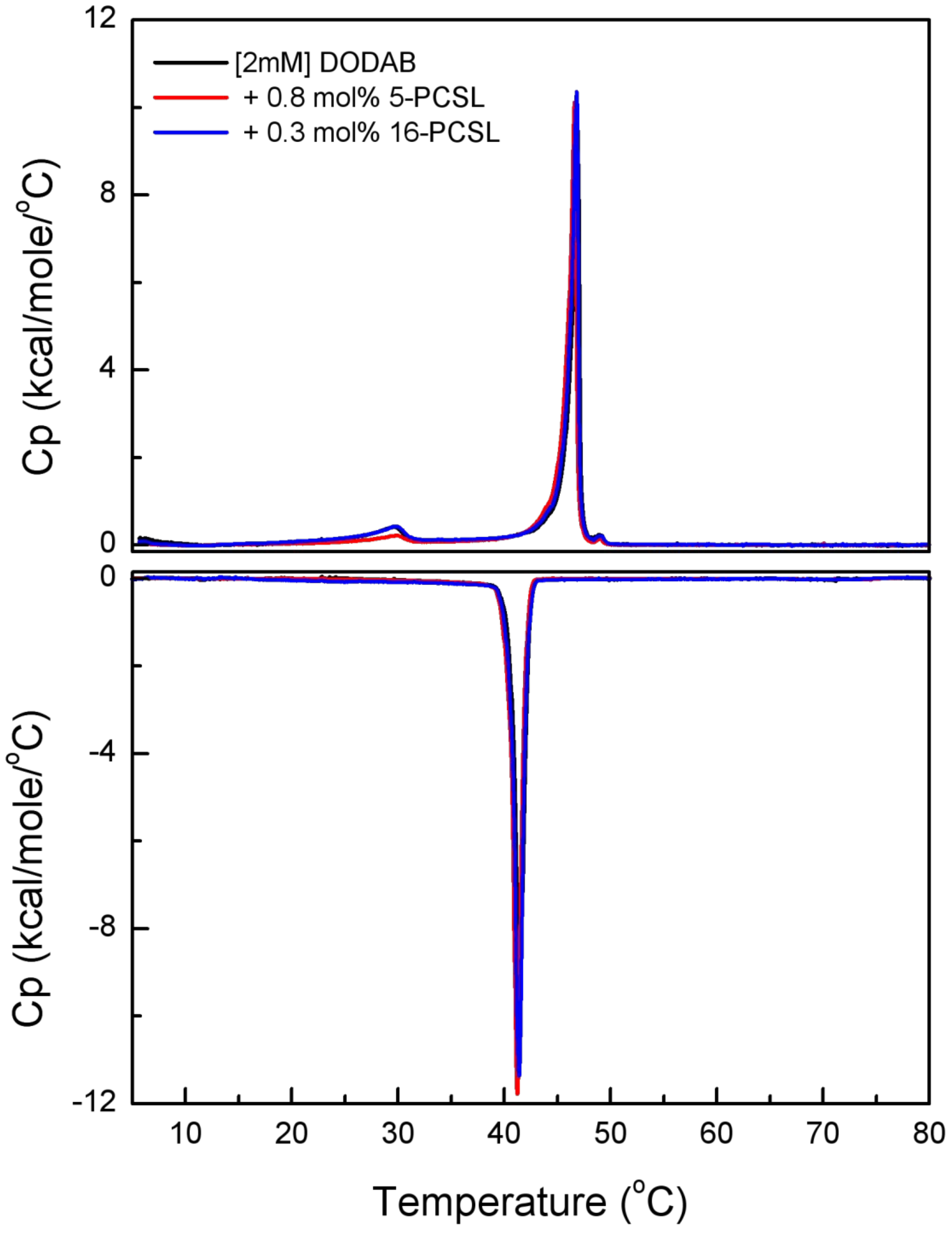

Supplementary Figure 1- Effect of 0.8 mol% 5-PCSL and 0.3 mol% 16-PCSL on the heating and cooling thermograms of DODAB vesicles. Scan rates were 20°C/hour.

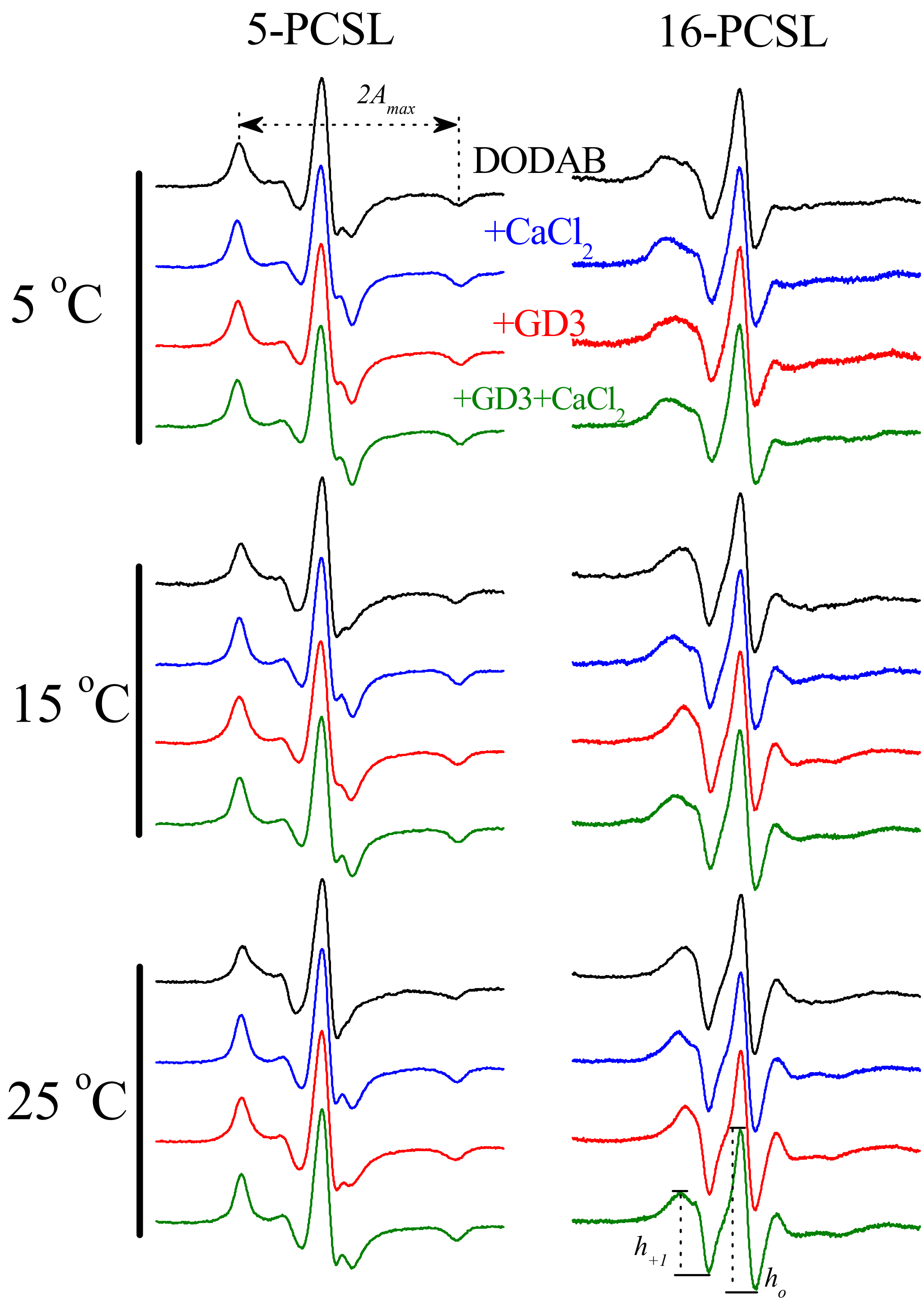

Supplementary Figure 2- EPR spectra of 5-PCSL and 16-PCSL embedded in 2 mM DODAB vesicles or in 2 mM DODAB + 0.1 mM GD3 vesicles, with or without 100 mM CaCl2, at temperatures below the gel-fluid transition. The maximum hyperfine splitting (A_{max}) and the amplitudes of low (h_{+1}) and central (h_0) field lines are indicated. The total spectra width is 100 G.
